# Evaluation and management of children with acute kidney injury in emergency department

**Published:** 2015-03-09

**Authors:** Abdolghader Pakniyat, Parsa Yousefichaijan

**Affiliations:** ^1^Student Research Committee, Emergency department, Arak University of Medical Sciences, Arak, Iran; ^2^Department of Pediatric Nephrology, Arak University of Medical Sciences, Arak, Iran

**Keywords:** Henoch-Schönlein purpura, Post infectious glomerulonephritis, Acute tubular necrosis

**Implication for health policy/practice/research/medical education:**

Acute kidney injury (AKI) is the abrupt loss of renal function and it is typically manifested by an increase in serum creatinine. The emergency physician must perform a full physical examination and obtain accurate medical history particularly nephrotoxic agents. Initially workup should include complete blood cell count; electrolyte, calcium, phosphorus, blood urine nitrogen, serum creatinine levels; urinalysis with microscopy and culture; chest x-ray; and renal ultrasonography. Patients with mild renal insufficiency due to pyelonephritis, Henoch-Schönlein purpura, post-infectious glomerulonephritis, or dehydration can managed on an outpatient basis but a nephrology consultation is necessary for fallow up but patients with hypertension, sever electrolytes abnormality, fluid over load must be hospitalized.

Acute kidney injury (AKI) is most important disorder in children who come to emergency department. Pathophysiology, epidemiology and treatment of children is different from adults due to differences in their anatomy and physiology.

AKI is the abrupt loss of renal function and it is typically manifested by an increase in serum creatinine. It is result of hypoxic and or nephrotoxic injury to the renal tubules and glomeruli. In the early stages of AKI with a reduced glomerular filtration rate (GFR) may have a relatively normal or slightly elevated creatinine, so early recognition and management of AKI are crucial (1,2). The major causes of AKI may be divided into prerenal; intrinsic renal; and post renal, although may migrate from one category to another; prerenal or post renal for an extended period may result in intrinsic renal damage and AKI. Prerenal physiology is not an uncommon finding in emergency departments and often due to gastrointestinal losses and acute tubular necrosis (ATN) is usually the consequence of hypo-perfusion (3). The patients can be asymptomatic, although almost of the patients were symptomatic consist of nausea, vomiting, diarrhea, history of recently post-streptococcal infection, bloody diarrhea, change in urine output and edema (2,3).

The emergency physician must perform a full physical examination and obtain accurate medical history particularly the medication used including herbal agents, sport supplements, non-steroidal anti-inflammatory drugs (NSAIDs), angiotensin-converting enzyme (ACE) inhibitors, angiotensin II receptor blockers (ARBs) and calcineurin inhibitors. Also the patient must be assessed accurately about the blood pressure, weight, hydration state, edema, skin manifestation, pulmonary and heart examination (3).

AKI laboratory finding including blood urea nitrogen (BUN) and creatinine rising, although the increase will not necessarily cause the creatinine to be more than normal range. The international classification system, Kidney Disease: Improving Global Outcomes (KDIGO) (Table 1), is preferred. The system uses creatinine and urine output criteria and can be applied to both children and adults, minimizing practice variation. Analysis of the urine can distinguish between prerenal, renal and post-renal causes of acute renal failure, although may be normal in prerenal AKI, so it is necessary to obtain urine specimen by catheterization in non-toilet trained children. Additionally, complete blood cell is useful to identify infection, hemolysis, anemia, thrombocytopenia and eosinophilia (2-4).

**Table 1:** KDIGO classification of renal injury

**Stage**	**Laboratory criteria**	**Urine output criteria**	**Other criteria**
**I**	Serum creatinine 1.5–1.9 times baseline or increase of ≥ 0.3 mg/dl	Urine output <0.5 ml/ kg/h for 6 h	
**II**	Serum creatinine 2–2.9 times baseline	Urine output <0.5 ml/kg/h for 12 h	
**III**	Serum creatinine 3 times baseline or increase in serum creatinine to ≥4 mg/dl	Urine output <0.3 ml/kg/h for 24 h or anuria for ≥12 h	Initiation or renal replacement therapy

Abbreviation: KDIGO, Kidney Disease: Improving Global Outcomes.

Electrolyte abnormalities is common in AKI due to renal function loss and decrease tubular secretion, cellular breakdown, dehydration and or volume overload. Electrolyte abnormality such as hyperkalemia, hyper or hyponatremia, etc, required emergent treatment, hence an ECG must be performed initially in all suspected AKI to identify cardiac arrhythmias. Other laboratory studies are dictated by the clinical feature (3).

The necessity imaging in patient with AKI in emergency department are; (*a*) Chest x-ray to identify cardiac size and pulmonary edema, (*b*) Renal ultrasonography, and (*c*) Voiding cystourethrography in boys with suspected posterior urethral valve (3,4).

Initially patients with suspected AKI must be monitored regarding vital sign, baseline weight and urine output. A bolus of 20 cc/kg crystalloid should be given in dehydration patients and blood transfusion must be considered in cases with hemorrhagic shock. If no urinary response is obtained after two boluses of crystalloid, diuretics is choice in euvolemic patient (Table 2) (4).

**Table 2:** Administration of drug agents in euvolemic patient with prerenal AKI

**Drug**	**Dose**	**Indication**	**Contraindication**
Furosemide	1 mg/kg per dose every 2 to 6 hours intravenously	No urinary response is obtained after two boluses of crystalloid	Urinary obstruction
Bumetanide	0.015 to 0.1 mg/kg per dose every 6 to 24 h intravenously (maximum 10 mg per day)	Furosemide has no effect	Urinary obstruction
Mannitol	0.75 g/kg per dose every 6 h intravenously		Urinary obstruction
Dopamine	2-5 µg/kg	No urine output despite diuretic therapy	

Abbreviation: AKI, acute kidney injury.

AKI may led to seizures fallowing either hypertensive encephalopathy or a metabolic derangement. Identified cause and treatment must be performed in children with AKI and all nephrotoxic agents must be limited or adjusted based on GFR, also the hypertension must be treated by use of nitroprusside or other intravenous blood pressure agents but not use ACEI, ARB and diuretics. In postrenal AKI Foley catheter maybe required to relive the obstruction, hydration therapy and treatment of hypertension is important and urologic consultation must be performed before discharging (4,5).

Finally, patients with mild renal insufficiency due to pyelonephritis, Henoch-Schönlein purpura, post-infectious glomerulonephritis or dehydration can be managed on an outpatient basis, however a nephrology consultation is necessary for fallow up, while patients with hypertension, sever electrolytes abnormality, fluid over load must be hospitalized (3).

## Authors’ contribution

PY and AP wrote the paper equally.

## Conflicts of interest

The authors declared no competing interests.

## Ethical considerations

Ethical issues (including plagiarism, data fabrication, double publication) have been completely observed by authors.

## Funding/Support

No funding from any source is directly associated with this manuscript.
